# Adaptation analysis of different noninvasive ventilation interfaces in critically ill patients

**DOI:** 10.1186/cc10149

**Published:** 2011-06-22

**Authors:** RMD Silva, KT Timenetski, RCM Neves, LH Shigemichi, SS Kanda, CC Rodrigues, RA Caserta, E Silva

**Affiliations:** 1 Hospital Israelita Albert Einstein, São Paulo - SP, Brazil

## Introduction

Noninvasive ventilation is a safe and effective method to treat acute respiratory failure, minimizing the respiratory workload and oxygenation. Few studies compare the efficacy of different types of noninvasive ventilation interfaces and their adaptation.

## Objective

To identify the most frequently noninvasive ventilation interfaces used and eventual problems related to their adaptation in critically ill patients.

## Methods

We conducted an observational study, with patients older than 18 years old admitted to the intensive care and step-down units of the Albert Einstein Jewish Hospital that used noninvasive ventilation. We collected data such as reason to use noninvasive ventilation, interface used, scheme of noninvasive ventilation used (continuously, periods or nocturnal use), adaptation, and reasons for nonadaptation.

## Results

We evaluated 245 patients with a median age of 82 years (range of 20 to 107 years). Acute respiratory failure was the most frequent cause of noninvasive ventilation used (71.3%), followed by pulmonary expansion (10.24%), after mechanical ventilation weaning (6.14%) and sleep obstructive apnea (8.6%). The most frequently used interface was total face masks (74.7%), followed by facial masks in 24.5% of the patients, and 0.8% used performax masks. The use of noninvasive ventilation for periods (82.4%) was the most common scheme of use, with 10.6% using it continuously and 6.9% during the nocturnal period only. Interface adaptation occurred in 76% of the patients; the 24% that did not adapt had their interface changed to improve adaptation afterwards. The total face mask had 75.5% of interface adaptation, the facial mask had 80% and no adaptation occurred in patients that used the performax mask. The face format was the most frequent cause of nonadaptation in 30.5% of the patients, followed by patient's related discomfort (28.8%), air leaking (27.7%), claustrophobia (18.6%), noncollaborative patient (10.1%), patient agitation (6.7%), facial trauma or lesion (1.7%), type of mask fixation (1.7%), and 1.7% patients with other causes.

## Conclusion

Acute respiratory failure was the most frequent reason for noninvasive ventilation use, with the total face mask being the most frequent interface used. The most common causes of interface nonadaptation were face format, patient-related discomfort and air leaking, showing improvement of adaptation after changing the interface used.

